# Distinct group, distinct traits? A comparison of risk factors across cybercrime offenders, traditional offenders and non-offenders

**DOI:** 10.1080/13218719.2025.2546311

**Published:** 2025-10-06

**Authors:** L. M. J. Bekkers, A. Moneva, E. R. Leukfeldt

**Affiliations:** ^a^ Center of Expertise Cyber Security, The Hague University of Applied Sciences, The Hague, Netherlands; ^b^ Netherlands Institute for the Study of Crime and Law Enforcement (NSCR), Amsterdam, Netherlands; ^c^ Leiden University, Leiden, Netherlands

**Keywords:** crime prevention, criminal behaviour, criminogenic needs, cybercrime, offending, risk factors

## Abstract

Individual differences associated with cybercrime offending remain under-researched. To address gaps in the literature and inform intervention, the current study compares cybercrime offenders, traditional offenders, and non-offenders in terms of risk factors for offending and online situational influences. We construct a survey using previously validated measurements, completed by 1,240 Dutch panel members aged 16 to 25. Two multinomial logistic regression models are estimated separately for cyber-dependent offenders and financial cybercrime offenders. Analysis reveals that cybercrime offenders overlap but also differ from traditional offenders. Cyber-dependent offenders seem to be a particular distinct offender group, while financial cybercrime offenders more closely resemble traditional offenders. Factors related to school, work, and leisure, but also personality, manifest differently for cybercrime: what was typically considered protective against traditional offending, such as higher levels of self-control, may actually provide opportunities to engage in cybercrime. Further longitudinal and experimental research is needed to support these conclusions.

## Introduction

Society is digitising. Nowadays, social traffic is routed through digital utilities and online platforms, which greatly impact our daily lives. Unfortunately, crime has also developed along that line. Digitisation offers unprecedented criminal opportunities and has made it possible for anyone to become involved in crime without physical convergence with victims or targets in space and time (Grabosky, 2003; Yar, 2005). This resulted in new types of crime that are conducted by means of information technology (IT), also referred to as cybercrime (Holt & Bossler, 2014; Maimon & Louderback, 2019; McGuire & Dowling, 2013a, 2013b).

A coherent definition and classification that accurately reflects the broad nature of cybercriminal behaviours is still lacking (Phillips et al., 2022). Two of the main cybercrime categories are cyber-dependent offences – that is, crimes that are fully conducted with and aimed at IT (Loggen et al., 2024a; McGuire & Dowling, 2013a) – and cyber-enabled financial-economic offences – that is, crimes that are facilitated by the use of IT but aimed at people for financial gain (Loggen et al., 2024b; McGuire & Dowling, 2013b). Among others, the first category refers to malware attacks (i.e. using malicious software to cause harm or damage to a computer system) and distributed denial-of-service attacks (DoS/DDoS; i.e. targeting the capacity of online services and network devices). The latter category includes financial offences such as online fraud (Cross, 2019). Nowadays, cyber-dependent crime and financial cybercrime belong to the most prevalent offences among civilians and businesses and are thought to impact victims heavily, not only financially but also emotionally (Borwell et al., 2022; Statistics Netherlands, 2023a).

Of course, the emergence of these kinds of criminal behaviours raises fundamental questions about their aetiology and the characteristics of people who engage in them (Dupont & Holt, 2023; Holt, 2023; Holt & Bossler, 2014, 2015; Leukfeldt & Holt, 2019). Are we dealing with a new type of offender that is associated with unique pathways and involvement mechanisms, or does cybercrime merely reflect a change in medium (the classic paradigm of ‘old wine in new bottles’; Grabosky, 2003)? Are theories that were originally developed for offline crimes applicable to online crimes as well? Such questions have major implications for our understanding of criminal conduct as well as offender prevention and rehabilitation.

That is, as formulated by the risk–need–responsivity (RNR) model, intervention can only be successful in reducing recidivism if intervention intensity matches the likelihood of offending of the target group, is focused on the factors that increase the likelihood of offending in the specific case of the target group, and is adjusted to learning styles and competencies of the target group (Andrews et al., 2006; Bonta & Andrews, 2007, 2023). Interventions are to be developed based on individual assessments of these risk, need and responsivity factors. In order to so, a thorough understanding is required of the behaviours, thoughts, feelings and social contexts of the people involved in crime.

Unfortunately, only few have focused on the distinguishing traits and developmental trajectories of cybercrime offenders (see Dietrich et al., 2016; Fox & Holt, 2021; Harbinson & Selzer, 2019; Holt et al., 2012; Holt et al., 2010; Loggen et al., 2024a, 2024b; Weulen Kranenbarg et al., 2017, 2018a, 2018b, 2019, 2022; Wissink et al., 2023), and existing studies have some important limitations. There is a lack of quantitative data, the application of standardised measures is rare, and most studies rely on convenience samples while being narrow in focus and examining a limited set of factors or offending behaviours. Also, a comprehensive understanding of the role of the ‘Central Eight’ risk factors of recidivism in cybercrime offending, even though these tend to be the best predictors of criminal behaviour overall (Andrews et al., 2006; Bonta & Andrews, 2007, 2023), is lacking. This means that current risk assessment instruments are insufficient to screen and measure the characteristics of cybercrime offenders. In turn, interventions are poorly adjusted to this heterogeneous target group, and are therefore limited in efficacy or may even have negative outcomes (Andrews & Dowden, 2007; Van der Wagen et al., 2021).

In an attempt to fill gaps in literature, the aim of the current study is to provide more insight into the defining risks and needs of cybercrime offenders. More specifically, we compare cyber-dependent offenders and financial cybercrime offenders with non-offenders and traditional offenders in terms of risk factors and online situational factors. We use survey data from 1240 Dutch youth. In the next section, we first discuss more in detail the individual differences associated with cybercrime offending in existing literature, after which we present the methodology and results of this study.

## Individual differences and cybercrime offending: a multi-theoretical approach

Over the last decades, researchers have coined many theories, paradigms and perspectives that aim to explain criminal behaviour (e.g. A. K. Cohen, 1955; Gottfredson & Hirschi, 1990; Merton, 1938; Sutherland et al., 1939). However, while this literature provides valuable insights, the General Personality and Cognitive Social Learning (GPCSL) perspective seems to prevail in terms of predictive quality across a wide range of different offending behaviours and target groups, considering years of scientific evidence (Bonta & Andrews, 2023).

In short, the GPCSL perspective proposes that people have a personality predisposition towards criminal behaviour, for instance in the form of low self-control, empathy deficits and negative emotionality. In addition to this, drawing on a rational choice perspective (Cornish & Clarke, 1986), it is presumed that an individual makes judgements about rewards and costs associated with criminal behaviours and prosocial behaviours: if benefits/rewards for criminal behaviour (e.g. financial profits) outweigh the benefits/rewards for prosocial behaviour (e.g. social approval of peers) and the costs of criminal behaviour (e.g. risk of being apprehended), then criminal behaviour is more likely to occur. This cognitive judgement is embedded within the specific social environment of an individual, meaning that criminal behaviour is learned through the interaction with others; people are more likely to exhibit delinquency if peers engage in such behaviours too. Finally, the immediate situational context in which the behaviour takes place is relevant in addition to individual propositions and characteristics, as it has a strong influence on the decision or intention of people to commit a crime. This is evident in the case that a burglar observes weak security in a car or house, for example.

In the GPCSL perspective, the major psycho-, social and biological risk factors for offending are translated into eight domains, hence they being called the ‘Central Eight’. These include (a) a history of offending, (b) antisocial personality patterns, (c) antisocial attitudes, (d) antisocial peer influences, (e) substance abuse, (f) family/martial circumstances, (g) school/work circumstances, and (h) leisure activities. Except for the first domain, which is a static risk factor, the Central Eight risk domains are criminogenic and changeable in nature. Changes in criminogenic factors directly relate to the likelihood of offending; the more the factors are present, the more likely a person is to commit crimes, and vice versa. Following the RNR-model, it is possible to alter the presence or expression of criminogenic needs, resulting in a lower likelihood of offending (Andrews et al., 2006; Bonta & Andrews, 2007, 2023). Thus, the distribution of the Central Eight risk/need factors can explain variability in criminal behaviour, and can guide towards effective offender prevention and rehabilitation (Bonta & Andrews, 2023). We now turn to current knowledge regarding the role of each of these risk domains in cybercrime offending.

Fox and Holt (2021) associated juvenile hacking with antisocial peer influences. Others also consistently relate exposure to delinquent peers to hacking (Holt et al., 2012; Walters, 2023), cyberstalking (Wissink et al., 2023), money muling (Bekkers et al., 2023) and media piracy (Holt et al., 2012), as well as general measures of cyberdeviance or cybercrime (Dearden & Parti, 2021; Holt et al., 2012). Next to the role of peers in cybercrime offending, low self-control is one of the most frequently mentioned explanations for various forms of cybercrime, as it would increase the likelihood that offenders act on opportunities to engage in crime while being online (Fox & Holt, 2021; Holt et al., 2012, 2021; Palmieri et al., 2021). The same goes for other deviant personality patterns such as psychopathy, although the role of these factors remains relatively understudied (Selzer & Oelrich, 2021). Similarly, techniques of neutralisation, in the literature used interchangeably with antisocial cognitions or pro-criminal attitudes, are considered to be one of key factors related to the onset and/or persistence of cybercrime behaviours, albeit most studies in this context focus on hacking and music piracy exclusively (Brewer et al., 2020; Chua & Holt, 2016; Holt et al., 2021; Walters, 2023). Delinquent peers, low self-control and neutralisation techniques were also mentioned to be key risk factors related to financial cybercrime offending in a systematic literature review (Loggen et al., 2024b).

Furthermore, some argue that substance abuse may be of less relevance in cyber-dependent offenders (Harbinson & Selzer, 2019), while it appeared to be a relatively important risk factor for other cybercrimes such as cyberstalking (Wissink et al., 2023). Drug addiction was also mentioned as a potential driver for financial cybercrime offending (Hutchings, 2013). There seems to be a role for family factors in the behaviour of cybercrime offenders as well. Aversive parental influences, such as parental separation or divorce, parental violence and low parental monitoring, relate to various cybercrime forms, including hacking and cyberstalking (Aborisade, 2023; Fox & Holt, 2021; Wissink et al., 2023). Additionally, it appears that cyber offenders in general are also more likely to have parents with a history of offending than non-offenders (Van de Weijer & Moneva, 2022).

As for work and school domains, it is mentioned that cybercriminal opportunities arising in professional life circumstances is one of the things distinguishing the life-course of cyber-dependent offenders from that of traditional offenders (Weulen Kranenbarg et al., 2018b, 2019, 2022). Here, it is argued that having a job or education that is unrelated to IT may reduce the odds of both cybercrime and traditional offending, perhaps due to increased social control (e.g. Weulen Kranenbarg et al., 2019). Similarly, higher levels of IT-skills were related to cyber-dependent offender groups, but not to traditional offender groups (Weulen Kranenbarg et al., 2018b). In the case of financial cybercrime, offending seems to be linked to lower levels of education (Loggen et al., 2024b). Finally, engaging in leisure activities, which is traditionally considered a protective factor against offending, may have different implications when considering the online environment. For instance, in a large longitudinal dataset it was found that engaging in offline leisure activities increased the likelihood of cybercrime offending in general, perhaps due to wider peer networks and exposure to deviant peer influences (Maras et al., 2024).

Next to the criminogenic need factors, there is also a role of the online environment as a direct facilitator of cybercriminal behaviours, similar to the physical environment influencing the decision to engage in offline crime. For instance, being online is associated with a state of disinhibition – for example, due to anonymity and a perceived lack of authority (Cheung et al., 2020; Kerstens & Jansen, 2016; Suler, 2004), which may increase the likelihood that people engage in illegal behaviours when presented with the opportunity. Similarly, Kerstens and Jansen (2016) link a lack of parental supervision and restriction regarding the online activities of their children to cybercrime perpetration. The hours spent online generally relate to an increased likelihood on various forms of cybercrime offending (Loggen et al., 2024a; Maras et al., 2024). That is, being online more often and engaging in gaming, forum use and social networking can expose young people to opportunities to offend in the online space (Kerstens & Jansen, 2016; Leukfeldt & Yar, 2016; Maras et al., 2024; Miró-Llinares & Moneva, 2019; Weulen Kranenbarg et al., 2019, 2022).

## Current study

This explorative survey study aims to compare the most common risk factors and online situational factors between young cybercrime offenders, traditional offenders and non-offenders (see also Weulen Kranenbarg, 2021). In the analysis, we distinguish between cyber-dependent offenders (i.e. crimes that are fully conducted with and aimed at IT) and financial cybercrime offenders (i.e. crimes that are conducted with IT but aimed at people for financial profits). We chose to focus on these offences because they belong to the most prevalent ones among youth in terms of victimisation and offending and have a high impact on victims and society at large (Borwell et al., 2022; Näsi et al., 2015; Statistics Netherlands, 2023a; Tollenaar et al., 2024). With this comparison and theoretical model, we gain more insight into the traits that define young people who commit cybercrime, as well as the role of direct and more distal online situational factors. This study adds to the body of knowledge on the applicability of criminological theory to cybercrime and on the individual differences associated with cybercrime offending, providing clinicians, probation officers, law enforcement and other relevant parties with insights for risk assessment and appropriate interventions.

## Method

### Procedure

Respondents were recruited via I&O Research, a research panel agency in the Netherlands. The panel consisted of 3419 Dutch youth. Data collection took place in August 2023. All panel members were notified about the study by email, with information about the study and its purpose and a link to an online questionnaire. The questionnaire started with an informed consent form, explaining the aim of the study and how the data were going to be handled. The panel members received a reminder to participate after one week and another one after two weeks. Those participating in the study received credits from the research agency, which they could convert into financial rewards or products. Respondents could pause and continue filling in the survey at any given time, resulting in completion times ranging from two minutes to 31,257 minutes (*M* = 1291.85; *SD* = 4203.42; *Mdn* = 20). This research was approved of by The Ethics Committee for Legal and Criminological Research (CERCO) and by the ethics review board of the Hague University of Applied Sciences. The research agency collected and stored the data in compliance with data regulations in the Netherlands, providing the researchers with an anonymised dataset containing no personal identifiable information.

### Respondents

After the two reminders, a total of 1,240 unique individuals aged between 16 and 25 participated in the survey, for a response rate of 36.3%. We filtered out speeders – that is, those respondents who completed the questionnaire in less than five minutes (*n* = 17). This left us with a sample of 1223 respondents. In this sample, there were more females (*n* = 837; 67.9%) than males (*n* = 396; 32.1%), while in the broader youth population (16–25 years) 50.8% are male, and 49.2% are female (Statistics Netherlands, 2023b), meaning that females were overrepresented in our sample. The same goes for level of education, as the majority of respondents currently followed or completed a study at university or higher professional education in the Netherlands (*n* = 805; 65.3%), in contrast to the broader Dutch population of individuals aged between 15 and 25 years, where around 14% have a high level of education (Statistics Netherlands, 2023c). Therefore, in order to increase representativeness, we used raking to weight the data according to youth population distributions on sex and level of education (Statistics Netherlands, 2023b, 2023c). Henceforth, we describe statistics based on weighted and filtered data.

Around half had a neighbourhood social-economic status (SES) below the country average (*n* = 541; 44.3%), and the other half above the country average (*n* = 681; 55.7%). SES-scores were determined in keeping with definitions of Statistics Netherlands (2023d) – for example, a score below −0.2 being the lowest, a score above 0.2 being the highest. Finally, most respondents had been previously victimised by some form of cybercrime in the past (*n* = 730; 59.7%).

### Measures

The online questionnaire combined validated screening measures derived from existing literature to assess offending behaviours, the Central Eight risk factors for recidivism and factors related to the online environment. In most cases, we used Dutch measures; if only English items or questionnaires were available in literature, the first author of this study, together with survey design experts from the research agency involved, translated the items to Dutch and then back to English for quality assurance. Constructs were calculated by summing the scores of the corresponding items. To control for social desirability, we administered the Dutch version of the Social Desirability Scale (SDS; Rutten et al., 2007) and included this measurement in the analysis.

#### Offending

##### Cybercrime offending

Respondents used dichotomous statements to indicate any past cybercrime offending behaviours. If so, an open-ended follow-up question appeared asking them to specify the frequency of this behaviour in the past 12 months. These items were derived from Weulen Kranenbarg et al. (2022), the Youth Delinquency Survey (YDS; Tollenaar et al., 2024; Van der Laan & Beerthuizen, 2021) and the International Self-Report Delinquency (ISRD4) study (Marshall et al., 2022). The cyber-dependent offences that we measured were: (a) technical hacking, (b) DoS/DDoS, (c) website defacement and (d) malware or viruses. The financial cybercrime offences that we measured were: (e) online fraud, (f) money muling, (g) virtual theft, (h) phishing, (i) bank helpdesk fraud and (j) online identity theft. Additionally, the survey measured a number of other cybercrime offences – namely, guessing or peeking passwords, image-based sexual abuse, online threats and online hate/discrimination. Respondents engaging in these offences were excluded from the non-offender group.

##### Traditional offending

Items on traditional offending were derived from the ISRD4 (Marshall et al., 2022) and the YDS (Tollenaar et al., 2024; Van der Laan & Beerthuizen, 2021) and included the following offences: (a) graffiti, (b) violence, (c) theft, (d) arson, (e) bike or motorcycle theft, and (f) vandalism. Items were presented and answered in similar fashion as the cybercrime offending behaviours, meaning we measured lifetime prevalence as well as frequencies of the behaviours in the past 12 months.

##### Cybercrime victimisation

Next to offending, the survey contained a number of items regarding previous cybercrime victimisation – namely: (a) hacking (not specified), (b) malware, (c) sexting, (d) online fraud, and (e) online identity fraud. These crimes belong to the most prevalent ones among youth in terms of victimisation (Näsi et al., 2015; Statistics Netherlands, 2023a). Items were derived from the study of Van’t Hoff-de Goede et al. (2019).

#### Central Eight risk domains

##### The Dark Triad

By means of the 12-item self-report questionnaire the Dirty Dozen (Jonason & Webster, 2010; Dutch translation from Klimstra et al., 2014,^1^) we measured the Dark Triad personality traits: psychopathy, narcissism and Machiavellianism. Participants responded with ‘1 = not at all’ to ‘9 = very much’ – for example, ‘I tend to manipulate others to get my way’ (Machiavellianism), ‘I tend to be cynical’ (psychopathy), and ‘I tend to want others to admire me’ (narcissism). The three subscales showed a Cronbach’s alpha of .72 or higher in previous research among adolescents and adults (Jonason & Luévano, 2013; Jonason & Webster, 2010: Klimstra et al., 2014). Higher scores corresponded to higher levels of Dark Triad traits.

##### Low self-control

The Brief Self-Control Scale (BSCS; Tangney et al., 2004) measures general self-control. On a 5-point scale, ranging from ‘1 = not at all like me’ to ‘5 = very much like me’, respondents rated how well 13 statements describe them (e.g. ‘I am lazy’). The BSCS previously demonstrated a Cronbach’s alpha of .85 or higher in samples of offenders and young people in school (Malouf et al., 2014; Pechorro et al., 2021). Items were coded in a way that a higher score implied lower levels of self-control.

##### Antisocial attitudes

The Criminal Sentiments Scale – Modified (CSS–M; Simourd & Van De Ven, 1999; α = .75), utilising a total of 41 items, measures four different subscales related to antisocial attitudes, beliefs and values associated with criminal behaviour – namely, Attitudes Towards the Law (e.g. ‘The law is rotten to the core’), Attitudes Towards the Court (e.g. ‘Judges are honest and kind’), Attitudes Towards the Police (e.g. ‘The police almost never help people’), Tolerance for Law Violations (e.g. ‘All laws should be obeyed’) and Identification with Criminal Others (e.g. ‘No one who breaks the law can be my friend’). Participants responded with ‘Agree’, ‘Not agree/Not disagree’ or ‘Disagree’. The higher the score, the more antisocial attitudes were present.

##### Antisocial peer influences

*Peer delinquency* were measured with the Measures of Criminal Attitudes and Associates (MCAA) scale (Mills et al., 2002). This scale showed very high reliability in a sample of young people (α > .90; O’Hagan et al., 2019). First, respondents were asked to think about the four key persons in their lives, after which they answered four statements about each of these persons with either ‘yes’ or ‘no’ (e.g. ‘Has person #1 ever been in jail?’). The second part of the MCAA consists of 46 items (‘agree’ or ‘disagree’) divided in four subscales, but for the purposes of this study, considering other measures were in place for criminal attitudes, we utilised eight items that related to the domain of criminal associates specifically (e.g. ‘I always feel welcomed around criminal friends’). The higher the scores, the more respondents were exposed to delinquent peers. We also asked respondents about *online deviant peer support* from online peers specifically with three items based on Yoon (2011) – for example, ‘My online friends and acquaintances would look down on me if I commit cybercrime’. Higher scores indicated greater exposure to online peers that support or encourage cybercrime offending.

##### Substance abuse

Respondents answered eight questions about their drug and alcohol use (richtlijnenjeugdhulp.nl) – for example, ‘Within the previous 12 months, have you felt irritated because people commented on your drug use?’ – with either ‘yes’ or ‘no’. A higher score meant greater substance abuse issues.

##### Family factors

The battery contained two dichotomously answered items related to the family circumstances of the respondent (e.g. ‘Did you ever witness a serious conflict or physical fight between your parents?’). These were derived from the YDS (Tollenaar et al., 2024; Van der Laan & Beerthuizen, 2021) and Fox and Holt (2021).

##### School factors

The battery contained two dichotomously answered questions about school commitment: ‘Have you ever been suspended from school’ and ‘Have you ever repeated a year in school?’. These items were derived from Fox and Holt (2021) and the YDS (Tollenaar et al., 2024; Van der Laan & Beerthuizen, 2021). We also asked respondents whether they currently or in the past had *worked in IT* or *followed education in IT*. Finally, we measured the subjective level of *IT-skills* of the respondents with one item derived from Weulen Kranenbarg et al. (2018b) and Holt et al. (2012). This is an ordinal measure, a so called Guttman-scale, in which respondents indicate which statement applies best to themselves: ‘1 = I don’t like using computers and don’t use them unless I absolutely have to’ to ‘5 = I can use different programming languages and am capable of detecting programming errors’. Although self-reported, in a previous study this scale correlated strongly to objective measurements of IT-skills (Weulen Kranenbarg et al., 2018b).

##### Leisure factors

With items derived from the YDS (Tollenaar et al., 2024; Van der Laan & Beerthuizen, 2021) and the Level of Service Inventory–Revised (LSI–R), and using a 5-point scale (‘1 = never’ to ‘5 = very often’), we asked respondents about participation in a number of leisure activities, including ‘How often do you participate in football or another ball sport?’ and ‘How often do you go shopping?’.

#### Online situational factors

##### Online disinhibition

This construct was measured with three items from Kerstens and Jansen (2016), such as ‘It is easier to express myself on the internet than in real life’. Response options ranged on a 7-point Likert-scale from ‘1 = totally disagree’ to ‘7 = totally agree’. The original study consists of six items, which showed good reliability among youth (α = .85; Kerstens & Jansen, 2016), but for survey length reasons we subtracted only three. Online disinhibition can be considered a state factor that varies throughout situations (Cheung et al., 2020), because of which we operationalise this construct within the realm of online situational factors. A higher score corresponded to higher levels of online disinhibition.

##### Online routine activities

We asked respondents how much time they spend on certain activities on the internet in an average week (Weulen Kranenbarg et al., 2018b) – namely, gaming, programming and communicating online. Items were answered on a 5-point scale: ‘1 = 0 hours’ to ‘5 = 21 hours or more’).

##### Online parental supervision

With three items on a 5-point Likert scale (1 = strongly disagree’ to ‘5 = strongly agree’) from Weulen Kranenbarg et al. (2022) and one item from the ISRD4 (Marshall et al., 2022), we asked respondents about the degree to which their parents supervise their activities on the internet and on computers – for example: ‘My parents know which websites and apps I use’. The higher the score, the more respondents perceived parental supervision on online activities.

### Analytical strategy

Data were analysed using SPSS Statistics Version 28. To provide more insight into the defining risks and needs of young cybercrime offenders, we used multinomial regression analyses, in which we compared cybercrime offenders with traditional offenders and non-offenders on the various crime predictors. We included cyber-dependent offenders and financial cybercrime offenders in separate models. Respondents who did not fall into any of the compared offender groups were excluded from the analysis. We chose to present the weighted models as the main analysis. We report the odds ratios (OR) as a measure of effect size and corresponding confidence interval (CI), in which values of 1.680, 3.470 and 6.710 correspond to Cohen’s *d* values of small (0.2), medium (0.4) and large (0.8) effect sizes, respectively (Chen et al., 2010). The performance of the models is measured by means of Nagelkerke *R*-squared, which is generally considered an informative coefficient in the case of regression analysis (Chicco et al., 2021). We used respondents who committed only traditional crimes in order to prevent overlap in the groups, but due to low amounts of respondents who committed only specific cybercrimes it was not meaningful to compare cybercrime groups with each other. With the groups that we used, we met recommendations of Bijleveld et al. (2018) on sample size for logistic regression analyses – that is, 10 observations per variable. Note that the cybercrime offender groups are not mutually exclusive: it is possible that the same respondent may have reported committing cyber offences from different categories. More information on mixed offending and specialisation is detailed in Appendix A. Finally, social desirability was included in the group comparisons as a control variable.

## Results

In this section, we first turn to the descriptives and psychometric properties related to our study variables (section ‘Descriptives and psychometric properties of study variables’). We then describe the prevalence of the different offending behaviours in our sample, listing both the unweighted and weighted outcomes (section ‘Prevalences of offending behaviours’). This is followed by the results of the multinomial logistic regression analysis with cyber-dependent offenders in the first group comparison (section ‘Cyber-dependent offenders: group comparison’) and financial cybercrime offenders in the second group comparison (section ‘Financial cybercrime offenders: group comparison’). The unweighted regression models are listed in Appendix B.^2^

### Descriptives and psychometric properties of study variables

Table 1 presents an overview of the constructs used as independent variables in the study and their internal consistencies. All of these variables meet common thresholds of .70 for acceptable internal consistencies in terms of Cronbach’s alpha (Nunnally & Bernstein, 1994), except for antisocial attitudes. Considering that this is an exploratory study, and the value is only slightly under the threshold, we chose to include the construct of antisocial attitudes in the analysis as is and recommend future research to use other measurements to study cognitions related to crime and deviance among cybercrime offenders. For continuous variables the table displays means and standard deviations, and for dichotomous variables the table displays the number of positive events. Note that scores on most of the categorial variables were unevenly distributed with the number of positive events being relatively rare.

**Table 1.** Overview of descriptives and psychometric properties of independent variables.

Variables	Minimum	Maximum	*M*	‘Yes’	*SD*	No. of items	Alpha
Continuous variables							
The Dark Triad	12	93	32.78	—	14.18	12	.864
Low self-control	13	65	35.52	—	9.41	13	.841
Antisocial attitudes	20	59	36.14	—	6.77	41	.650
Peer delinquency	0	12	1.43	—	2.11	24	.757
Online deviant peer support	3	15	4.23	—	2.45	3	.894
Substance abuse	0	8	0.61	—	1.19	8	.718
IT-skills	1	5	2.65	—	0.99	1	—
Time spent shopping	1	5	2.73	—	0.79	1	—
Time spent at sports	1	5	1.91	—	1.22	1	—
Online disinhibition	3	15	5.96	—	3.32	3	.777
Time spent gaming	1	5	2.14	—	1.14	1	—
Time spent programming	1	5	1.23	—	0.70	1	—
Time spent communicating online	1	5	3.13	—	1.06	1	—
Online parental supervision	4	20	9.66	—	3.74	4	.744
Dichotomous variables							
Suspended from school	0	1	—	68	—	1	—
Redid school year	0	1	—	326	—	1	—
IT-work	0	1	—	69	—	1	—
IT-education	0	1	—	97	—	1	—
Witnessed parental violence	0	1	—	189	—	1	—
Was under supervision of youth care	0	1	—	76	—	1	—

*Note: n* = 1223.

### Prevalences of offending behaviours

Table 2 depicts the self-reported offending behaviours of the respondents. Our offence classification is in line with existing literature (e.g. Loggen et al., 2024a, 2024b; McGuire & Dowling, 2013a, 2013b).

**Table 2.** Self-reported prevalence of offending behaviours.

	Prevalence(unweighted)			Prevalence(weighted)		
	*n*	*%*	*SD*	*n*	*%*	*SD*
Cyber-dependent crime						
Technical hacking	16	1.3	0.113	9	0.7	0.084
DoS/DDoS	6	0.5	0.070	10	0.8	0.091
Website defacement	18	1.5	0.120	28	2.3	0.149
Malware or viruses	3	0.2	0.049	1	0.1	0.030
* Total*	37	3	0.171	46	3.7	0.190
Financial cybercrime						
Online fraud	2	0.2	0.040	5	0.4	0.062
Phishing	2	0.2	0.040	6	0.5	0.071
Bank helpdesk fraud	1	0.1	0.028	0	—	0.017
Virtual theft	48	3.9	0.194	52	4.3	0.203
Money muling	6	0.5	0.070	14	1.2	0.107
Online identity theft	26	2.1	0.144	30	2.5	0.155
* * * Total*	73	5.9	0.236	88	7.2	0.258
Traditional crime						
Graffiti	88	7.1	0.258	99	8.1	0.273
Violence towards people	17	1.4	0.117	17	1.4	0.117
Theft	299	24.2	0.429	302	24.7	0.431
Bike or motorcycle theft	13	1.1	0.102	15	1.2	0.109
Vandalism	34	2.8	0.164	41	3.4	0.180
Arson	89	7.2	0.259	113	9.2	0.290
* Total traditional offenders*	410	33.3	0.471	429	35.1	0.477
* * * Total traditional only offenders*	249	20.2	0.402	244	20	0.400

*Note: n* = 1223. DoS/DDoS = distributed denial-of-service attacks.

A total of 334 respondents (27.3%) reported to have committed at least one form of cybercrime at some point in the past, while 429 respondents (35.1%) reported a history of traditional offending. Regarding cybercrime, after applying the weights, the most prevalent offence was non-technical hacking (*n* = 223; 18.2%), followed by online hate/discrimination (*n* = 84; 6.8%) and virtual theft (*n* = 52; 4.3%). Also, respondents seem to engage in financial offending generally at a higher rate than the cyber-dependent offences. With regard to traditional crimes, crimes with a high self-reported prevalence were theft (*n* = 302; 24.4%) and graffiti (*n* = 104; 8.4%). The prevalence of self-reported cybercrime and traditional crime in the current study were both higher than those from the YDS (Tollenaar et al., 2024), but the prevalence of cybercrime was lower in the current study than findings of Weulen Kranenbarg et al. (2022).

As for offender groups, we identified 46 cyber-dependent offenders (i.e. committed at least one cyber-dependent offence at some point, regardless of any other offending behaviours), 88 financial cybercrime offenders (i.e. committed at least one financial cybercrime at some point, regardless of any other offending behaviours), 244 traditional only offenders (i.e. committed at least one traditional offence at some point, but did not commit a cybercrime offence) and 644 non-offenders (i.e. not involved in any offence that was measured in the survey). These groups are included in the multinominal regression models. Note that some respondents may thus belong to more than one cybercrime category. For more information about crime specialisation among participants we refer to Appendix A.

### Cyber-dependent offenders: group comparison

Table 3 describes the results of the group comparisons between cyber-dependent offenders (*n* = 46), traditional only offenders (*n* = 244) and non-offenders (*n* = 644). With regard to the criminogenic risk domains, cyber-dependent offenders scored higher than non-offenders on peer delinquency, online deviant peer support and substance abuse, had higher self-control, were more likely to have a job in IT and were more likely to have been under supervision of child care. Non-offenders tended to game more often, while cyber-dependent offenders came from neighbourhoods with a higher social-economic status.

**Table 3.** Multinomial logistic regression model listing, *OR* and CI for cyber-dependent offenders.

	Model 1		Model 2	
Comparison group	Cyber-dependent offenders		Cyber-dependent offenders	
Reference group	Non-offenders		Traditional offenders	
*Central Eight domains*	*OR*	95% CI	*OR*	95% CI
Antisocial personality patterns:				
The Dark Triad	1.022	0.988–1.057	1.006	0.972–1.040
Low self-control	0.934*	0.881–.990	0.926*	0.873–0.982
Antisocial attitudes	0.973	0.908–1.043	0.990	0.922–1.063
Antisocial peer influences:				
Peer delinquency	1.515**	1.242–1.849	1.223*	1.008–1.484
Online deviant peer support	1.543**	1.317–1.807	1.394**	1.188–1.635
Substance abuse	1.746**	1.259–2.421	1.514*	1.095–2.093
School/work factors:				
Been suspended (yes)	2.032	0.405–10.204	1.471	0.319–6.776
Redid school year (yes)	0.736	0.252–2.149	0.609	0.206–1.803
Job in IT (yes)	Positive**	3.674–87.374	Positive**	3.301–96.906
Education in IT (yes)	0.434	0.088–2.125	0.306	0.058–1.617
IT-skills	0.737	0.450–1.207	0.780	0.471–1.291
Family factors:				
Witnessed parental violence (yes)	1.243	0.375–4.122	1.090	3.590
Under supervision childcare (yes)	4.666*	1.142–19.063	2.147	0.537–8.579
Leisure factors:				
Time spent shopping	0.981	0.569–1.691	1.302	0.746–2.273
Time spent at ball sports	0.974	0.696–1.365	0.811	0.578–1.139
*Online situational factors*				
Online disinhibition	0.958	0.828–1.108	0.995	0.859–1.151
Online routine activities:				
Time spent gaming	0.561*	0.351–.899	0.726	0.450–1.170
Time spent programming	1.402	0.815–2.411	1.786*	1.002–3.184
Time spent communicating online	1.085	0.716–1.644	0.899	0.591–1.367
Online parental supervision	0.917	0.810–1.038	0.934	0.823–1.060
*History and demographics*				
Neighbourhood SES	1.317*	1.022–1.699	1.470*	1.135–1.903
Age	0.665	0.415–1.065	0.638	0.395–1.031
Level of education (low)	0.459	0.135–1.563	0.496	0.141–1.743
Sex (female)	0.525	0.186–1.479	0.462	0.162–1.321
Previous victimisation (yes)	1.200	0.457–3.155	0.682	0.254–1.831
Nagelkerke *R^2^*	0.328			
McFadden *R^2^*	0.196			
Cox–Snell *R^2^*	0.256			
Likelihood ratio test	276.065**			
Akaike Information Criterion (AIC)	1241.724			
Bayesian Information Criteria (BIC)	1503.083			
*N*	934			

*Note:* SES = socioeconomic status; *OR* = odds ratio; CI = confidence interval.

*p < .05. **p < .001.

Compared to traditional offenders, cyber-dependent offenders scored higher on peer delinquency, online deviant peer support, substance abuse and having a job in IT, were more likely to spend time programming, and had higher neighbourhood SES-scores, while traditional offenders were characterised by lower self-control. Also, although results were not significant, an interesting observation is that the cyber-dependent offenders tended to be predominantly males.

Do note that in some cases CIs were large, especially having a job in IT, probably resulting from small sample sizes of cyber-dependent offenders. The large CIs lower the precision of our findings, decreasing the chances to detect meaningful relationships as statistically significant. Although the relationships for job in IT were significant and in a positive direction (23.9% of the cyber-dependent offenders had a job in IT compared to 4% of non-offenders and 3.7% of the traditional offenders), we removed the specific values from the table since they can be considered unreliable. The coefficients of determination of this model imply that the independent variables together are able to explain some of the variation in cyber-dependent offending: Nagelkerke pseudo *R^2^* = .328; McFadden *R^2^* = .196; Cox-Snell *R^2^* = .256. The current model showed the best fit in terms of Akaike information criterion (AIC) and Bayesian information criterion (BIC) values.

### Financial cybercrime offenders: group comparison

Table 4 describes the results of the group comparisons between financial cybercrime offenders (*n* = 88), exclusive traditional offenders (*n* = 244) and non-offenders (*n* = 644). As for the Central Eight domains, financial offenders scored higher than non-offenders on the Dark Triad traits, peer delinquency and online deviant peer support. They were also more likely to have been suspended from school, to have experienced substance abuse and to have been under supervision of child care, but were less likely to engage in shopping. Furthermore, it appeared that financial cybercrime offenders were more likely to exhibit a lack of parental supervision online, to live in neighbourhoods with a high SES-score and to have been victimised by cybercrime.

**Table 4.** Multinomial logistic regression model listing, *OR* and CI for financial cybercrime offenders.

	Model 1		Model 2	
Comparison group	Financial cybercrime offenders		Financial cybercrime offenders	
Reference group	Non-offenders		Traditional offenders	
*Central Eight domains*	*OR*	95% CI	*OR*	95% CI
Antisocial personality patterns:				
The Dark Triad	1.041**	1.018–1.064	1.024*	1.002–1.048
Low self-control	0.977	0.942–1.013	0.964	0.928–1.001
Antisocial attitudes	1.016	0.973–1.062	1.036	0.989–1.085
Antisocial influences:				
Peer delinquency	1.428**	1.259–1.619	1.173*	1.036–1.329
Online deviant peer support	1.301**	1.158–1.461	1.177*	1.043–1.328
Substance abuse	1.374*	1.062–1.779	1.198	
School/work factors:				
Been suspended (yes)	3.543**	1.194–10.512	2.761*	1.008–7.564
Redid school year (yes)	0.770	0.385–1.540	0.627	0.307–1.279
Job in IT (yes)	2.629	0.755–9.151	2.100	0.526–8.387
Education in IT (yes)	0.716	0.215–2.388	0.641	0.172–2.383
IT-skills	1.410	0.982–2.027	1.467*	1.010–2.129
Family factors:				
Witnessed parental violence (yes)	0.777	0.354–1.705	0.659	0.301–1.447
Under supervision childcare (yes)	3.641*	1.340–9.891	1.889	0.694–5.141
Leisure factors:				
Time spent shopping	0.637*	0.432–0.938	0.854	0.571–1.276
Time spent doing ball sports	0.815	0.628–1.057	0.706	0.542–0.918
*Online situational factors*				
Online disinhibition	0.932	0.847–1.025	0.979	0.888–1.078
Online routine activities:				
Time spent gaming	1.192	0.914–1.554	1.446*	1.090–1.919
Time spent programming	0.663	0.419–1.048	0.969	0.594–1.582
Time spent communicating online	1.003	0.753–1.337	0.825	0.615–1.107
Online parental supervision	0.831**	0.760–0.908	0.849**	0.774–0.931
*History and demographics*				
Neighbourhood SES	1.343**	1.132–1.593	1.490**	1.248–1.779
Age	0.780	0.574–1.061	0.763	0.554–1.052
Level of education (low)	0.786	0.322–1.916	0.875	0.344–2.222
Sex (female)	1.420	0.730–2.763	1.188	0.597–2.363
Previous victimisation (yes)	2.027*	1.047–3.926	1.154	0.577–2.308
Nagelkerke *R^2^*	0.340			
McFadden *R^2^*	0.193			
Cox–Snell *R^2^*	0.276			
Likelihood ratio test	315.927**			
Akaike Information Criterion (AIC)	1427.626			
Bayesian Information Criteria (BIC)	1691.366			
*N*	977			

*Note:* SES = socioeconomic status; *OR* = odds ratio; CI = confidence interval.

*p < .05. **p < .001.

Compared to traditional offenders, financial cybercrime offenders showed higher levels of Dark Triad traits, peer delinquency and online deviant peer support. Financial cybercrime offenders also had more IT-skills, spent less time at sports, were more likely to have been suspended from school, lacked parental supervision, had higher SES-scores and spent more time gaming. This model was associated with the following coefficients of determination: Nagelkerke pseudo *R^2^* = .340; McFadden *R^2^* = .193; Cox–Snell *R^2^* = .276. These values are similar to the cyber-dependent offender model.

## Discussion

In the current exploratory survey study, we compared cyber-dependent offenders and financial cybercrime offenders with traditional offenders and non-offenders on traditional risk factors and online situational factors (see also Weulen Kranenbarg, 2021). The survey consisted of previously validated instruments and screened for offending behaviours and key risk factors of crime, as also found in forensic populations and the most common risk assessment instruments. This survey was administered among youth panel members from a renowned research agency in the Netherlands and completed by a sample of 1240 Dutch individuals aged between 16 and 25 years. Multinomial logistic regression analysis was used to compare the different groups on the study variables. With this unique, multi-theoretical approach, we address important gaps and methodological limitations of current literature (see, for instance, Loggen et al., 2024a, 2024b) and can provide better insights into the factors that contribute specifically to cybercrime offending.

Generally speaking, it seems that cybercrime offending can be attributed partially to key criminogenic risk domains, similar to traditional offending (Andrews et al., 2006; Bonta & Andrews, 2007, 2023). However, we did find important differences between the groups on a number of points.

As for the cyber-dependent offenders – for example, those involved in technical offences such as website defacement and DDoS attacks – it appeared that this group scored higher than non-offenders on delinquent peers and online deviant peer support, had higher self-control, had higher levels of substance abuse, were more likely to have a job in IT, were less likely to game and were more likely to have been under supervision of child care. Cyber-dependent offenders also came from neighbourhoods with a higher social-economic status. Having a job in IT was one of the key distinguishing factors. Compared to traditional offenders, similar results were found overall, with cyber-dependent offenders also being more likely to engage in programming.

These findings partly contradict traditional criminological insights. For instance, cyber-dependent offenders were more likely to live in prosperous neighbourhoods, to display higher levels of self-control and to pursue jobs in information technology. The more technologically sophisticated offences may require patience, time and effort to get the hang of it, which may not be realistic for those with low self-control (Bossler & Burruss, 2011; Fox & Holt, 2021; Holt & Bossler, 2015). This opposes the general theory of crime (Gottfredson & Hirschi, 1990) and implies that different developmental pathways may play a role for this offender group. What is also particularly relevant for cyber-dependent offending is the role of both online and offline peer environments (e.g. Dearden & Parti, 2021). That is, our study indicates that opportunities for cyber-dependent crimes arise out of peer contact and are embedded within deviant social contexts (Dearden & Parti, 2021; Holt, 2020; Holt & Bossler, 2015; Holt et al., 2012; Hutchings, 2014; Hutchings & Clayton, 2016). Antisocial associates may be accessed via jobs related to IT (e.g. Weulen Kranenbarg et al., 2019), where young people become part of social clusters in which it is normalised to experiment on the Internet and explore the possibilities of technology (Kerstens & Jansen, 2016; Weulen Kranenbarg et al., 2019, 2022). Here, peers may provide the encouragements, neutralisations and justifications to further escalate in cybercrime behaviours.

Findings related to gaming are also in contrast to previous literature that identified gaming as a key pathway into cyber-dependent crimes (e.g. Hutchings, 2014; Loggen et al., 2024b; National Crime Agency, 2017; Wall, 2017): non-offenders were more likely to game. Generally speaking, gaming can possibly redirect young people who are interested and skilled in technology away from deviant behaviours to more prosocial leisure activities. Perhaps there are specific forms of gaming that would pose a risk – for example, gaming done together with peers – which can potentially expose young people to deviant influences. The current study did not differentiate in types of gaming. More research on this topic is thus required, preferentially with a larger sample of cyber-dependent offenders and a more specified measurement of gaming.

These findings have important implications for intervention and prevention. Engaging in jobs or educations related to IT – for example, as part of resocialisation and rehabilitation programmes for cybercrime offenders (see, for instance, Schiks et al., 2024) – can possibly have adverse effects due to exposure to like-minded peers. Similarly, what was thought to constitute protective personality patterns may actually provide opportunity to engage in cybercrime. We recommend further study of pathways into cybercrime using qualitative methodologies, for instance by means of offender interviews or police file analysis, and to utilise prospective longitudinal data. Noticeable here is the trade-off between offline environments and cybercrime: peers in the physical world relate to online offending. This means that cybercrime does not merely exist online and that such offences are still dependent on offline mechanisms.

As for the financial cybercrime offenders, what seems to distinguish this group from non-offenders are higher scores on Dark Triad traits and more exposure to antisocial influences in offline and online environments. Financial cybercrime offenders were also more likely to be suspended from school, to experience substance abuse, to have been under supervision of child care and to lack parental oversight online, while they were less likely to engage in shopping. Finally, financial cybercrime offenders were also more likely to be previously victimised by cybercrime and to live in neighbourhoods with a high social-economic status. Compared to traditional offenders, we found similar results with a number of significant differences disappearing.

In fact, financial cybercrime offenders generally showed more similarities with traditional offenders, exhibiting an individual profile that is more in line with expectations from traditional criminological literature (e.g. Andrews et al., 2006). For example, Dark Triad personality patterns related to financial cybercrime offending but not to cyber-dependent offending. Such deviant individual characteristics have received little attention in cybercrime offending literature (see also Selzer & Oelrich, 2021). Indeed, the majority of the financial cybercrime offenders were involved in traditional crimes as well, as depicted in Appendix B. In other words, factors that tend to predict offline offending may play a similar role in financial cybercrimes, although additional longitudinal research is needed to validate such statements. This means that insights derived from existing criminological literature can also be used to understand financial cybercrime offending and to intervene accordingly in the light of the RNR-model. However, differences were also observed, particularly related to the role of IT and online opportunities: financial cybercrime offenders had higher levels of IT-skills, lacked parental supervision online and spent more time gaming.

These findings indicate that the role of the online environment as a facilitator for cybercrime cannot be ignored, especially a lack of online parental supervision (e.g. Kerstens & Jansen, 2016). Such factors should perhaps be addressed in risk assessment efforts of cybercrime offenders and that online situational crime prevention strategies can possibly be effective in reducing online offending behaviours (Cornish & Clarke, 2003; Kerstens & Jansen, 2016; Weulen Kranenbarg et al., 2019, 2022). Situational crime prevention aims to prevent criminal behaviour by reducing opportunities to commit crime, a perspective that is applied more and more to cyberspace (e.g. Ho et al., 2022). From this perspective, it is key that parents or caregivers apply rules regarding Internet and computer usage of their children, and that children experience more monitoring and oversight on their online activities. This is in line with the concept of perceived guardianship, which is rooted in routine activity theory (L. E. Cohen & Felson, 1979; Leukfeldt & Yar, 2016) and the rational choice perspective (Cornish & Clarke, 1986): an absence of a capable guardian is one of the key factors that can explain criminal behaviour. Therefore, we encourage open communications between parents and their children about online experiences, interactions and discussions with peers, including potential risks. Parents should be provided with the necessary resources and information to conduct such conversations.

We did not statistically compare the cybercrime groups with each other due to overlapping respondents and few respondents specialising in a certain cybercrime (see Appendix A). We encourage future research endeavours to study this comparison using larger samples. Nonetheless, it seems that the groups have in common that they are influenced by peers, both online and offline, and also lack parental supervision online. Differences were observed as well. Having a job in IT and higher levels of self-control related specifically to cyber-dependent offending, while financial offenders were more similar to traditional offenders overall in terms of criminogenic needs. However, due to limitations of our data – for example, no information on temporal ordering of offences – we think it is important that future researchers focus on overlap between offending behaviours, on transitions from one type of crime to another and on escalation in seriousness of offending, in which longitudinal data would be key.

It is important to consider the findings of this study in the light of some limitations. Firstly, we screened for risk factors in non-clinical populations rather than doing actual risk assessment in forensic settings, and therefore measured only subclinical levels. This means our findings are exploratory in nature and should be treated as a first step towards better understanding the risks and needs of individuals involved in cybercrime. Additional research on incarcerated cybercrime offenders and using standardised risk assessment instruments is necessary to validate our findings. Secondly, we used respondents who only committed traditional crimes in the group comparisons in order to create mutually exclusive groups, while the cybercrime offender groups also contained mixed offenders. Because of this, the traditional offender group may reflect the less serious offenders (e.g. Rokven et al., 2018). Thirdly, as with any other self-report study on crime, there may have been social desirable responding or underreporting of offending behaviours, although we controlled for social desirability in the group comparisons by including this factor in the analysis as a variable. It can be beneficial to use police data or to study forensic populations in addition to survey research, in order to counter each other’s limitations, and to measure and study actual cybercrime offending.

Altogether, findings indicate that some premises and assumptions from traditional criminological theories may not hold up for cybercrime offending, especially in the case of cyber-dependent crimes (e.g. Burruss et al., 2019; Fox & Holt, 2021; Holt & Bossler, 2015; Maras et al., 2024; Rokven et al., 2018; Weulen Kranenbarg et al., 2019). This applies to the domains of school, work and leisure in particular, but also in terms of personality. That is, pursuing work in IT, having IT-skills and having higher levels of self-control may actually provide opportunities to engage in cybercrime, rather than being protective. This means that risk factors for traditional crimes may manifest themselves differently for cybercrimes, and that especially cyber-dependent offenders constitute a distinct offender group in terms of individual characteristics and situational influences (e.g. Rokven et al., 2018; Weulen Kranenbarg et al., 2019). This may call for revisions in risk assessment instruments for cybercrime offenders, although more research is required. Here, both research and practice should differentiate between various cybercrime groups, primarily based on the level of technological sophistication of offences.

## Supplementary Material

Supplementary material.docx

## Footnotes

1 The Dutch version was obtained directly via its authors since it was not published online.

2 Generally, relationships in the weighted and unweighted models were in the same direction, though the coefficients tended to be lower in magnitude. For instance, in the unweighted models, substance abuse did not significantly relate to the cybercrime groups, whereas it did in the weighted models.

## Appendix A. Crime specialization of offenders.

**Table:**

Offender group	*N*	Exclusive		Also cyber-dependent		Also financial cybercrime		Also traditional	
		*n*	*%*	*n*	*%*	*n*	*%*	*n*	*%*
Cyber-dependent offenders	46	17	37	—	—	23	50	19	41.3
Financial cybercrime offenders	88	16	18.2	23	26.1	—	—	56	63.6
Traditional offenders	429	244	56.9	19	4.4	56	13.1	—	—

## Appendix B. Unweighted regression models

**Table:**

	Model 1		Model 2	
Comparison group	Cyber-dependent offenders		Cyber-dependent offenders	
Reference group	Non-offenders		Traditional offenders	
*Central Eight domains*	*OR*	95% CI	*OR*	95% CI
Antisocial personality patterns:				
The Dark Triad	1.014	0.983–1.046	1.001	0.970–1.033
Low self-control	1.011	0.959–1.066	0.972	0.921–1.026
Antisocial attitudes	0.944	0.881–1.013	0.959	0.893–1.030
Antisocial peer influences:				
Peer delinquency	1.468**	1.227–1.756	1.097	0.928–1.297
Online deviant peer support	1.458**	1.246–1.705	1.340**	1.144–1.569
Substance abuse	1.326	0.960–1.833	1.156	0.840–1.591
School/work factors:				
Been suspended (yes)	1.616	0.210–12.465	0.799	0.124–5.158
Redid school year (yes)	0.632	0.224–1.781	0.516	0.182–1.461
Job in IT (yes)	3.104	0.764–12.618	3.810	0.859–16.898
Education in IT (yes)	2.623	0.596–11.554	1.814	0.382–8.620
IT-skills	0.964	0.602–1.543	0.853	0.529–1.378
Family factors:				
Witnessed parental violence (yes)	1.224	0.414–3.617	0.737	0.250–2.170
Under supervision childcare (yes)	2.880	0.674–12.312	2.438	0.585–10.157
Leisure factors:				
Time spent shopping	0.964	0.560–1.658	1.299	0.747–2.257
Time spent at ball sports	1.062	0.763–1.478	1.052	0.753–1.470
*Online situational factors*				
Online disinhibition	0.923	0.797–1.069	0.990	0.854–1.148
Online routine activities:				
Time spent gaming	0.782	0.527–1.160	0.920	0.617–1.374
Time spent programming	0.809	0.476–1.372	0.958	0.552–1.663
Time spent communicating online	0.888	0.591–1.333	0.765	0.507–1.154
Online parental supervision	0.938	0.832–1.057	0.947	0.839–1.070
*History and demographics*				
Neighbourhood SES	0.957	0.772–1.186	0.997	0.802–1.241
Age	0.748	0.460–1.217	0.719	0.438–1.180
Level of education (low)	0.632	0.223–1.795	0.616	0.214–1.774
Sex (Female)	0.357*	0.142–0.897	0.377*	0.148–0.963
Previous victimisation (yes)	1.661	0.694–3.974	1.286	0.528–3.133
Nagelkerke *R^2^*	.278			
McFadden *R^2^*	.164			
Cox–Snell *R^2^*	.214			
Likelihood ratio test	1144.531**			
Akaike Information Criterion (AIC)	1252.531			
Bayesian Information Criteria (BIC)	1513.689			
* N*	931			
Antisocial personality patterns:				
The Dark Triad	1.039**	1.016–1.061	1.024*	1.002–1.046
Low self-control	1.025	0.988–1.063	0.984	0.948–1.022
Antisocial attitudes	1.016	0.968–1.065	1.032	0.982–1.084
Antisocial peer influences:				
Peer delinquency	1.423**	1.244–1.627	1.065	0.944–1.203
Online deviant peer support	1.307**	1.153–1.483	1.200*	1.058–1.361
Substance abuse	1.254	0.982–1.602	1.095	0.861–1.393
School/work factors:				
Been suspended (yes)	3.652	0.963–13.851	2.123	0.659–6.835
Redid school year (yes)	0.704	0.339–1.461	0.545	0.261–1.141
Job in IT (yes)	1.837	0.536–6.300	2.200	0.576–8.405
Education in IT (yes)	0.519	0.129–2.099	0.412	0.095–1.791
IT-skills	1.374	0.963–1.959	1.195	0.831–1.718
Family factors:				
Witnessed parental violence (yes)	0.450	0.176–1.152	0.276*	0.109–0.699
Under supervision childcare (yes)	2.278	0.719–7.214	1.985	0.633–6.221
Leisure factors:				
Time spent shopping	0.694	0.467–1.030	0.943	0.627–1.418
Time spent at ball sports	0.835	0.635–1.098	0.843	0.638–1.115
*Online situational factors*				
Online disinhibition	0.990	0.901–1.088	1.063	0.965–1.171
Online routine activities:				
Time spent gaming	1.292	0.990–1.686	1.467*	1.110–1.940
Time spent programming	0.775	0.489–1.230	0.952	0.586–1.549
Time spent communicating online	1.025	0.988–1.063	0.898	0.668–1.205
Online parental supervision	0.936	0.859–1.019	0.941	0.861–1.027
*History and demographics*				
Neighbourhood SES	1.026	0.939–1.120	1.122	0.959–1.313
Age	0.853	0.605–1.204	0.815	0.571–1.162
Level of education (low)	0.799	0.390–1.636	0.796	0.381–1.664
Sex (female)	1.169	0.589–2.318	1.159	0.572–2.346
Previous victimisation (yes)	1.748	0.932–3.280	1.365	0.708–2.631
Nagelkerke *R^2^*	.294			
McFadden *R^2^*	.165			
Cox–Snell *R^2^*	.236			
Likelihood ratio test	1314.789**			
Akaike Information Criterion (AIC)	1422.789			
Bayesian Information Criteria (BIC)	1685.996			
* N*	967			

*Note:* SES = socioeconomic status; *OR* = odds ratio; CI = confidence interval.

**p* < .05. ***p* < .001.
