# Supplementary material for: Distinct group, distinct traits? A comparison of risk factors across cybercrime offenders, traditional offenders and non-offenders
Source: Psychiatr Psychol Law. 2025 Oct 6;33(5):1367–91. doi: 10.1080/13218719.2025.2546311 (PMC13618152; doi:10.1080/13218719.2025.2546311)
Supplement: Supplementary file 1 [file TPPL_A_2546311_SM8349.docx]

In these supplementary materials, we present multinomial regression models for non-technical hacking. Non-technical hacking refers to peaking or guessing passwords. The sample contained 223 non-technical hackers, i.e., respondents who indicated to have engaged in non-technical hacking next to any other offending behaviors. These models are not part of the main text because they are beyond the scope of the manuscript, but still relevant for readers. Information about how groups were constructed and how analysis was performed can be found in the main body of text.

*Table 1.* Multinomial logistic regression model listing OR and CI for non-technical hackers (weighted)

|  | **Model 1** | |  | | | | **Model 2** | | | |  | | |
| --- | --- | --- | --- | --- | --- | --- | --- | --- | --- | --- | --- | --- | --- |
| Comparison group | Non-technical hackers  Non-offenders | | | | | | Non-technical hackers  Traditional offenders | | | | | | |
| Reference group |  |  |  |  |  |  |  |  |  |  |  |  |  |
| *Central Eight domains* | *OR* | | *95%-CI* | | | | *OR* | | | | *95%-CI* | | |
| Antisocial personality patterns: |  | |  | | | |  | | | |  | | |
| The Dark Triad | 1.032** | | 1.017-1.047 | | | | 1.016* | | | | 1.000-1.032 | | |
| Low self-control | .990 | | .966-1.015 | | | | .978 | | | | .952-1.004 | | |
| Antisocial attitudes | .956* | | .929-.985 | | | | .970 | | | | .940-1.002 | | |
| Antisocial peer influences: |  | |  | | | |  | | | |  | | |
| Peer delinquency | 1.199** | | 1.090-1.317 | | | | .973 | | | | .891-1.063 | | |
| Online deviant peer support | 1.189 | | 1.097-1.289 | | | | 1.088 | | | | 1.000-1.183 | | |
| Substance abuse | 1.363** | | 1.147-1.621 | | | | 1.150 | | | | .974-1.357 | | |
| School/work factors: |  | |  | | | |  | | | |  | | |
| Been suspended (yes) | 2.209 | | .933-5.231 | | | | 1.788 | | | | .799-4.001 | | |
| Redid school year (yes) | .962 | | .447-1.072 | | | | .578* | | | | .361-.925 | | |
| Job in IT (yes) | 1.941 | | .744-5.066 | | | | 1.692 | | | | .547-5.233 | | |
| Education in IT (yes) | .741 | | .309-1.776 | | | | .605 | | | | .220-1.662 | | |
| Family factors: |  | |  | | | |  | | | |  | | |
| Witnessed parental violence (yes) | 1.546 | | .967-2.471 | | | | 1.267 | | | | .772-2.077 | | |
| Under supervision childcare (yes) | 1.122 | | .517-2.436 | | | | .580 | | | | .268-1.255 | | |
| Leisure factors: |  | |  | | | |  | | | |  | | |
| Time spent shopping | .827 | | .652-1.051 | | | | 1.093 | | | | .839-1.425 | | |
| Time spent at ball sports | .958 | | .819-1.121 | | | | .815* | | | | .689-.964 | | |
|  |  | |  | | | |  | | | |  | | |
| *Online situational factors* |  | |  | | | |  | | | |  | | |
| IT-skills | 1.172 | | .935-1.469 | | | | 1.251 | | | | .973-1.609 | | |
| Online disinhibition | .962 | | .904-1.024 | | | | 1.000 | | | | .935-1.069 | | |
| Online routine activities: |  | |  | | | |  | | | |  | | |
| Time spent gaming | .940 | | .780-1.133 | | | | 1.135 | | | | .920-1.402 | | |
| Time spent programming | .873 | | .623-1.224 | | | | 1.266 | | | | .851-1.885 | | |
| Time spent communicating online | 1.268* | | 1.064-1.510 | | | | 1.090 | | | | .901-1.318 | | |
| Online parental supervision | .898** | | .851-.947 | | | | .919* | | | | .865-.976 | | |
|  |  | |  | | | |  | | | |  | | |
| *History and demographics* |  | |  | | | |  | | | |  | | |
| Neighborhood SES | .986 | | .890-1.092 | | | | 1.071 | | | | .955-1.201 | | |
| Age | .842 | | .690-1.027 | | | | .847 | | | | .679-1.057 | | |
| Level of education (low) | .840 | | .498-1.419 | | | | .992 | | | | .552-1.783 | | |
| Sex (female) | 1.549* | | 1.023-2.346 | | | | 1.324 | | | | .841-2.083 | | |
| Previous victimization (yes) | 2.684** | | 1.811-3.978 | | | | 1.502 | | | | .962-2.344 | | |
|  |  | | |  | |  | | |  | | | | |
| Nagelkerke R^2^ | | .287 | | |  | | |  | | | |  |  |
| McFadden R^2^ | | .145 | | |  | | |  | | | |  |  |
| Cox-Snell R^2^ | | .246 | | |  | | |  | | | |  |  |
| Likelihood ratio test | | 313.374** | | |  | | |  | | | |  |  |
| Akaike Information Criterion (AIC) | | 1953.640 | | |  | | |  | | | |  |  |
| Bayesian Information Criteria (BIC) | | 2224.369 | | |  | | |  | | | |  |  |
| *N* | | 1,112 | | | |  | | | |  | | |  |

* p<.05; ** p<.001

*Table 1.* Multinomial logistic regression model listing OR and CI for non-technical hackers (unweighted)

|  | **Model 1** | |  | | | | **Model 2** | | | |  | | |
| --- | --- | --- | --- | --- | --- | --- | --- | --- | --- | --- | --- | --- | --- |
| Comparison group | Non-technical hackers  Non-offenders | | | | | | Non-technical hackers  Traditional offenders | | | | | | |
| Reference group |  |  |  |  |  |  |  |  |  |  |  |  |  |
| *Central Eight domains* | *OR* | | *95%-CI* | | | | *OR* | | | | *95%-CI* | | |
| Antisocial personality patterns: |  | |  | | | |  | | | |  | | |
| The Dark Triad | 1.026** | | 1.012-1.040 | | | | 1.012 | | | | .997-1.027 | | |
| Low self-control | 1.020 | | .998-1.043 | | | | .978 | | | | .954-1.003 | | |
| Antisocial attitudes | .979 | | .952-1.007 | | | | .994 | | | | .963-1.025 | | |
| Antisocial peer influences: |  | |  | | | |  | | | |  | | |
| Peer delinquency | 1.209** | | 1.092-1.338 | | | | .913* | | | | .834-1.000 | | |
| Online deviant peer support | 1.169** | | 1.068-1.280 | | | | 1.076 | | | | .981-1.181 | | |
| Substance abuse | 1.265** | | 1.085-1.475 | | | | 1.096 | | | | .937-1.282 | | |
| School/work factors: |  | |  | | | |  | | | |  | | |
| Been suspended (yes) | 2.794 | | .939-8.313 | | | | 1.564 | | | | .586-4.177 | | |
| Redid school year (yes) | .782 | | .507-1.206 | | | | .607* | | | | .382-.964 | | |
| Job in IT (yes) | 1.544 | | .667-3.575 | | | | 2.143 | | | | .779-5.901 | | |
| Education in IT (yes) | 1.291 | | .527-3.164 | | | | 1.003 | | | | .358-2.813 | | |
| Family factors: |  | |  | | | |  | | | |  | | |
| Witnessed parental violence (yes) | 1.630* | | 1.065-2.493 | | | | .959 | | | | .611-1.506 | | |
| Under supervision childcare (yes) | .677 | | .268-1.713 | | | | .623 | | | | .249-1.559 | | |
| Leisure factors: |  | |  | | | |  | | | |  | | |
| Time spent shopping | 1.010 | | .808-1.263 | | | | 1.354* | | | | 1.048 | | |
| Time spent at ball sports | 1.033 | | .896-1.191 | | | | 1.030 | | | | .876-1.211 | | |
|  |  | |  | | | |  | | | |  | | |
| *Online situational factors* |  | |  | | | |  | | | |  | | |
| IT-skills | 1.146 | | .927-1.416 | | | | 1.027 | | | | .807-1.307 | | |
| Online disinhibition | .986 | | .930-1.045 | | | | 1.054 | | | | .987-1.126 | | |
| Online routine activities: |  | |  | | | |  | | | |  | | |
| Time spent gaming | .977 | | .817-1.168 | | | | 1.123 | | | | .915-1.378 | | |
| Time spent programming | .750 | | .553-1.018 | | | | .879 | | | | .614-1.259 | | |
| Time spent communicating online | 1.156 | | .976-1.369 | | | | 1.015 | | | | .839-1.227 | | |
| Online parental supervision | .953 | | .906-1.002 | | | | .963 | | | | .910-1.020 | | |
|  |  | |  | | | |  | | | |  | | |
| *History and demographics* |  | |  | | | |  | | | |  | | |
| Neighborhood SES | .962 | | .880-1.052 | | | | .988 | | | | .892-1.095 | | |
| Age | .858 | | .704-1.047 | | | | .841 | | | | .670-1.055 | | |
| Level of education (low) | .734 | | .489-1.102 | | | | .778 | | | | .492-1.228 | | |
| Sex (female) | 1.127 | | .743-1.710 | | | | 1.133 | | | | .712-1.802 | | |
| Previous victimization (yes) | 1.741* | | 1.234-2.458 | | | | 1.360 | | | | .911-2.031 | | |
|  |  | | |  | |  | | |  | | | | |
| Nagelkerke R^2^ | | .233 | | |  | | |  | | | |  |  |
| McFadden R^2^ | | .114 | | |  | | |  | | | |  |  |
| Cox-Snell R^2^ | | .201 | | |  | | |  | | | |  |  |
| Likelihood ratio test | | 255.157** | | |  | | |  | | | |  |  |
| Akaike Information Criterion (AIC) | | 2093.476 | | |  | | |  | | | |  |  |
| Bayesian Information Criteria (BIC) | | 2364.475 | | |  | | |  | | | |  |  |
| *N* | | 1,138 | | | |  | | | |  | | |  |

* p<.05; ** p<.001
